# Features of the organization of bread wheat chromosome 5BS based on physical mapping

**DOI:** 10.1186/s12864-018-4470-y

**Published:** 2018-02-09

**Authors:** Elena A. Salina, Mikhail A. Nesterov, Zeev Frenkel, Antonina A. Kiseleva, Ekaterina M. Timonova, Federica Magni, Jan Vrána, Jan Šafář, Hana Šimková, Jaroslav Doležel, Abraham Korol, Ekaterina M. Sergeeva

**Affiliations:** 1 Institute of Cytology and Genetics, Siberian Branch, Russian Academy of Sciences, Novosibirsk, Russia; 2 University of Haifa, Haifa, Israel; 3 Istituto di Genomica Applicata, Udine, Italy; 4 Institute of Experimental Botany, Centre of the Region Haná for Biotechnological and Agricultural Research, Olomouc, Czech Republic

**Keywords:** Chromosome 5BS, Hexaploid wheat, *Triticum aestivum*, Genetic markers, Physical mapping, Sequencing, Synteny

## Abstract

**Background:**

The IWGSC strategy for construction of the reference sequence of the bread wheat genome is based on first obtaining physical maps of the individual chromosomes. Our aim is to develop and use the physical map for analysis of the organization of the short arm of wheat chromosome 5B (5BS) which bears a number of agronomically important genes, including genes conferring resistance to fungal diseases.

**Results:**

A physical map of the 5BS arm (290 Mbp) was constructed using restriction fingerprinting and LTC software for contig assembly of 43,776 BAC clones. The resulting physical map covered ~ 99% of the 5BS chromosome arm (111 scaffolds, N50 = 3.078 Mb). SSR, ISBP and zipper markers were employed for anchoring the BAC clones, and from these 722 novel markers were developed based on previously obtained data from partial sequencing of 5BS. The markers were mapped using a set of Chinese Spring (CS) deletion lines, and F2 and RICL populations from a cross of CS and CS-5B *dicoccoides*.

Three approaches have been used for anchoring BAC contigs on the 5BS chromosome, including clone-by-clone screening of BACs, GenomeZipper analysis, and comparison of BAC-fingerprints with in silico fingerprinting of 5B pseudomolecules of *T. dicoccoides.* These approaches allowed us to reach a high level of BAC contig anchoring: 96% of 5BS BAC contigs were located on 5BS. An interesting pattern was revealed in the distribution of contigs along the chromosome. Short contigs (200–999 kb) containing markers for the regions interrupted by tandem repeats, were mainly localized to the 5BS subtelomeric block; whereas the distribution of larger 1000–3500 kb contigs along the chromosome better correlated with the distribution of the regions syntenic to rice, Brachypodium, and sorghum, as detected by the Zipper approach.

**Conclusion:**

The high fingerprinting quality, LTC software and large number of BAC clones selected by the informative markers in screening of the 43,776 clones allowed us to significantly increase the BAC scaffold length when compared with the published physical maps for other wheat chromosomes. The genetic and bioinformatics resources developed in this study provide new possibilities for exploring chromosome organization and for breeding applications.

**Electronic supplementary material:**

The online version of this article (10.1186/s12864-018-4470-y) contains supplementary material, which is available to authorized users.

## Background

Physical mapping as an approach aimed at revealing the order of extended DNA regions on chromosomes has undergone several changes associated with advances in genetic, genomic, and cytological methods for the analysis of genome organization. At the very beginning, restriction mapping of the whole genome with subsequent electrophoretic analysis modified for extended DNA regions was used to examine the small genomes of protists and fungi [1, 2]. However, longer genomes required preliminary fragmentation of chromosomes using BAC and YAC vectors [3, 4]. The cloned genome regions are arranged along the chromosome relative to each other either according to cytological mapping, using the BioNano Genome Mapping platform [5], or according to high-throughput fingerprinting using SNaPshot or whole genome profiling (WGP) technologies [6, 7]. These approaches aim at achieving the so-called “gold standard” for quality reference sequences of eukaryotic genomes.

Wheat is interesting firstly in studying the reorganization of a large eukaryote genome in the process of allopolyploidization, the interaction of genes and their activation or suppression in response to the change in copy number, and to study the contribution of natural and artificial selection to the formation of adaptive features. Secondly, the information obtained by studying the structural and functional organization of the genome finds rapid application in breeding strategies of this highly important food crop.

However, the large size of the wheat genome (17 Gb), and the abundance of different families of repeated DNA sequences, with no less than a threefold repetition of genetic loci, create considerable impediments for the rapid decoding of the genome. To overcome these difficulties, a consortium for the collaborative sequencing of the wheat genome was organized, with the basic strategy of chromosomal division of the genome, followed by cloning of each individual chromosome or chromosome arm in a BAC library with reasonable coverage and final construction of physical maps for subsequent sequencing. The first pilot project to build a physical map of chromosome 3B, with a length of 960 Mbp, was completed in 2008 [8]. Such an approach based on the construction of physical maps via BAC cloning, is rather laborious, time-consuming and expensive. Not surprisingly, the emergence of Next Generation Sequencing (NGS) methods in 2005, which enable substantial reductions in time and cost, has led to a significant increase in the number of projects on whole genome sequencing and re-sequencing, including organisms with a large genome size (more than 1 Gb) [9].

In 2012, sequencing of the 17-gigabase-pair wheat hexaploid genome using 454 pyrosequencing, was first reported. Although the genome coverage was 5-fold only and N50 for the assembled reads was only 481 bp, it enabled the identification of ~ 96,000 genes, and defined a genome-wide catalogue of single nucleotide polymorphisms (SNPs) in the A, B and D genomes for future genetic and genomic studies [10]. The first works that enabled a chromosome-based draft of the hexaploid bread wheat (*Triticum aestivum*) genome to be obtained using NGS were published in 2014 [11]. Sequencing of Chinese Spring (CS) individual chromosomes sorted by flow-cytometry was carried out to a depth of between 30× and 241× with Illumina technology platforms. As a result, 61% of the wheat genome sequence was assembled, with the L50 of repeat-masked assemblies ranging from 1.7 to 8.9 kb. The next round of NGS sequencing of the wheat genome, bypassing physical mapping, was aimed at improving the assembly and annotation of the allohexaploid wheat genome, relative to the previously obtained data for individual CS chromosomes [11] and for the genome of a synthetic wheat line, W7984 [12]. For this, a new wheat whole-genome shotgun sequencing with coverage of more than 33× was undertaken. For assembly, a combination of optimized data types and assembly algorithms were designed and employed to deal with the large and complex genomes [13]. As a result, the new assembly represents > 78% of the genome, and the contiguity of the assembly as measured by scaffold N50 values is 3.7-fold greater than that of the synthetic wheat assembly [12] and 30 times that of the CS assembly [11]. Thus, improvements in approaches to sequencing and software aimed at assembling the genome have significantly improved the quality of the genome assembly.

It is worth noting that the reference genome of wild tetraploid wheat was constructed based on a combination of whole-genome shotgun (WGS) and sequencing of various insert-size libraries, with genetic data and three-dimensional (3D) chromosome conformation capture sequencing (HiC) data [14]. The sequencing and assembly approaches used resulted in a three-fold increase of scaffold N50 values (57,378 bp), relative to the latest work performed on the genome of hexaploid wheat [13]. As a result, a 10.1-gigabase assembly of the 14 chromosomes was obtained, which represents > 84% of the wheat tetraploid genome [14].

Despite such outstanding success in wheat whole genome sequencing through NGS bypassing BAC cloning, a “gold standard” reference sequence of the allopolyploid wheat genome was not obtained. Physical mapping using BAC cloning is still the only approach that enables this “gold standard” of reference sequence to be obtained for the common wheat genome. Furthermore, precision in construction of the physical map, which involves various approaches for anchoring BAC-contigs, is especially important to accelerate map-based cloning, especially for genes from regions where recombination is reduced or absent. To reach the “gold standard” of the wheat genome reference sequences, the International Wheat Genome Sequencing Consortium (IWGSC; http://www.wheatgenome.org) uses a “chromosome-based strategy”, including the physical mapping of individual chromosomes as one step in this approach.

In the framework of IWGSC, our aim is to develop and use the physical map for analysis of the short arm of wheat chromosome 5B (5BS). This arm of the wheat chromosome bears a number of agronomically important genes, such as the crossability gene (*Skr*), grain softness protein (Gsp-B1), genes connected to resistance to fungal diseases *(Lr52, LrK1, Yr47, Snn3, Pm 30,* QTL for stem rust); genes affecting morphological and physiological traits such as canopy temperature depression, copper efficiency, pre-harvest sprouting and hairy peduncles [15–18].

This physical map will be a significant step for map-based gene cloning and provide a foundation for improving agricultural traits in crop breeding programs. The length of the short arm of chromosome 5B is 290 Mb. Like other chromosomes of the B-genome, 5BS has several large heterochromatin blocks represented by tandem repeats, the largest of them in the subtelomeric and near-centromere regions. The high level of heterochromatization of the short arm of chromosome 5B required the involvement of various approaches in constructing a good quality physical map.

## Methods

### Plant material and DNA extraction

The CS-5Bdic disomic substitution line, where the Chinese Spring (CS) 5B chromosome is replaced by the *T. dicoccoides* 5B chromosome (5Bdic) was kindly provided by Prof. B.S. Gill (Kansas State University, Manhattan, USA) and J.D. Faris (Agricultural Research Center, Fargo, ND 58105, USA). The F2 population of the cross between CS and CS-5Bdic disomic substitution line, comprising 366 individuals, was developed for genetic mapping. We also used а population of 116 recombinant inbred chromosomal lines (RICLs) obtained after seven generations of self-pollination of the F2 population of CS x CS-5Bdic, and genotyped using the Illumina Infinium 15 k Wheat platform (TraitGenetics GmbH; [19]).

Cytogenetic mapping of molecular markers was performed using: (i) The N5BT5A nullitetrasomic line (in which 5B chromosome is replaced with 5A), (ii) N5BT5D nullitetrasomic line (5B chromosome is replaced with 5D), (iii) Dt5BL ditelosomic line (lacking 5BS chromosome arm), and (iv) a set of 10 CS chromosome 5B deletion lines (http://www.k-state.edu/wgrc/genetic_resources/chinese_spring_deletion_lines.html Germplasm/Deletions/del_index.html). The nullitetrasomic lines were used to confirm localizations of markers and genes in the 5B chromosome. The ditelosomic line Dt5BL was used to localize markers on the short or long arms. Deletion homozygous CS lines confirmed by SSR markers [20] were used for localization of markers on deletion bins (C-5BS3–0.41, 5BS3–0.41-0.42, 5BS2–0.42-0.43, 5BS4–0.43-0.56, 5BS8–0.56-0.71, 5BS1–0.67-0.71, 5BS5–0.71-0.81, and 5BS6–0.81-1.00).

Works on the development and reproduction of wheat lines were carried out at the Joint Access Center for Artificial Plant Cultivation ICG SB RAS.

### Marker development and analysis

Sequences specific to the 5BS chromosome obtained earlier [21] were used to design TE junction–based markers. The ISBP (insertion site–based polymorphism) markers were developed with the ISBP Finder script [22]. Primer sequences for the designed ISBPs are presented in Additional file 1. We also used published SSR markers available in the GrainGenes database (https://wheat.pw.usda.gov/GG3/), in our previously publication [23] and the newly designed SSR markers for 5BS, included in this study (Additional file 1).

### Construction of the 5BS BAC library

The DNA samples for the short arm of CS chromosome 5B were purified by flow cytometric sorting from the CS 5B double ditelosomic line (2*n* = 40 + 2t5BS + 2t5BL). The seeds were kindly provided by Prof. A. Lukaszewski (University of California, Riverside, USA). Liquid suspensions of intact mitotic metaphase chromosomes were prepared from synchronized meristem root tips of young seedlings according to [24]. The chromosome suspensions were stained with DAPI (2 μg/ml) and analyzed at a rate of 1500–2000 events/s in a FACSAria II SORP flow sorter (BD Biosciences, San José, USA), whereas DNA for the chromosome arm was sorted at a rate of 20 events/s. Sorted fractions were tested for contamination by other chromosomes. Samples of 1000 chromosomes sorted onto a microscope slide were analyzed by fluorescence in situ hybridization (FISH) with a probe for the GAA microsatellite [25].

BAC libraries specific for 5BS were constructed according to [26]. This approach comprised the following steps: partial DNA digestion with *Hin*dIII; size selection by pulsed-field gel electrophoresis; electroelution of 100–200-kb DNA fragments from the gel followed by ligation into the *Hin*dIII-digested dephosphorylated pIndigoBAC-5 vector (Epicentre, Madison, USA); and transformation of *Escherichia coli* DH10B T1 competent cells (Invitrogen, Carlsbad, USA) with the recombinant vector. The 5BS-specific BAC library (TaaCsp5BShA) was arrayed in 114 plates (384 wells each) and stored at − 80 °C; the wells contained freezing medium 2YT, 6.6% glycerol, and 12.5 μg/ml chloramphenicol. In total, 43,776 clones were obtained for the short arm of chromosome 5B. Analysis of randomly selected BAC clones gave an average insert size of 122 kb.

### BAC library fingerprinting

The BAC DNA samples of the 5BS-specific library were isolated using the Agencourt CosMCPrep BAC Purification kit (Beckman Coulter) followed by labeling and fingerprinting according to the HICF SNaPshot protocol [27] modified for the physical maps of hexaploid wheat [8]. The BAC fingerprint profiles (peak areas, peak heights, and fragment sizes) were obtained by capillary electrophoresis in an ABI 3730 sequencer. Fingerprint background (vector, low signal, and partially digested peaks) and cross-contamination were removed using GeneMapper (http://www.lifetechnologies.com) and FPB software [28]; the band sizes for each BAC clone were formatted and united into a single list to fit the FingerPrinted Contig (FPC) input data format.

### Contig assembly and MTP selection

Computational assembly of high-quality BAC fingerprints was performed using the FingerPrinted Contigs (FPC) [29] and Linear Topological Contig (LTC) [30] programs. The automatic FPC assembly procedure was conducted according to the protocol approved for wheat physical mapping [8]. Initial assembly of 32,283 5BS clones was performed at a Sulston score of 10^− 75^, followed by stepwise merging by increasing the cut-off by 10^− 5^ at each step to the final merge at 10^− 45^. After each merging, the contigs with more than 10% of questionable (Q) clones were broken up using the DQer function and reassembled with an increasing cut-off stringency of up to 10^− 84^. Finally, the contigs were merged end-to-end in an automated mode using a cut-off of 10^− 45^ or higher and at least 50 common bands in ending clones. Minimum Tilling Path (MTP) clones from FPC assembly were selected with the following parameters: Min FPC overlap Sulston score 1e^− 30^, Min dist from previous MTP BAC end = 30 bands, and Min shared bands between the adjacent MTP clones = 12.

LTC assembly of 5BS was performed using the parameters described by Raats et al. [31]. Significant clone overlaps were arranged into a network using a Sulston score cut-off of 10^− 15^. A cut-off of 10^− 15^ was used to exclude Q-overlaps and a cut-off of 10^− 25^, to exclude Q-clones. To split a large group of highly overlapping clones, a cut-off of 10^− 30^ was used to exclude an additional set of Q-clones. Groups of overlapping clones were split into contigs with linear topology using another set of Q-clones excluded manually. The clones with over 500 highly significant (< 10–50) overlaps were excluded as well. LTC contigs were assembled automatically with a stringency cut-off of 10^− 15^ after the Q-clones and Q-overlaps were excluded.

MTP clones for LTC contigs were obtained using the LTC program with a natural requirement of a significant clone overlap for adjacent MTP clones (at a cut-off of 10^− 15^ to 10^− 33^ corresponding to approximately 30–50% of shared bands depending on the clone length). Reliable LTC based contigs were end-to-end merged into scaffolds via less reliable (unproven by parallel clone overlaps) clone and clone overlaps [31].

### Validation of physical map data

Physical scaffolds constructed by LTC consist of reliable parts (with coverage > 3, physical contigs) connected via a single BAC or single pair of overlapping clones. To avoid false connections (due to false significant clone overlaps or chimerical clones caused by DNA contamination) we compared positions of markers (SSR and ISBP) on physical and on genetic maps. This comparison helps validate the physical map via the following: (a) The presence of the same marker at the ends of two physical scaffolds provides additional argument for end-to-end merging of these scaffolds; (b) Presence of a marker in the set of overlapping BACs in the internal part of the contig and in a single clone from another place in the physical map indicates that this single clone is presumably a chimerical clone; (c) Presence of a marker in two or more groups of overlapping clones from different internal parts of physical contig(s) shows that the marker is not unique; (d) Presence of markers that belong to the same physical scaffold but are separated by a considerable distance on the genetic map indicates inconsistency between the genetic and physical map. If the corresponding positions on the genetic map are well proven then such physical scaffolds should be split. This approach was also used for several other wheat chromosome arms, e.g., 1BS [31], 1BL [32], 5AS and 5AL [33].

### IonTorrent and BAC-end sequencing

BAC clones from the middle part of contigs were sequenced using two approach. BAC DNA was isolated using NucleoSpin 96 Flash (Macherey-Nagel) kit. The sequencing on the Ion Torrent platform and assembled with the MIRA program were described previously [23].

For BAC-end sequencing, 5 μl of purified BAC DNA (~ 0.2 to 0.5 μg) was used in a sequencing reaction with ABI BigDye terminators (Applied Biosystems, Foster City, CA). Template DNA was sequenced from both directions with pCC1BAC/pIndigoBAC-5 Forward and Reverse End-Sequencing Primers (Epicentre, Madison, WI). Electrophoresis of the sequencing reaction was carried out with a 3730xl DNA Analyzer (Applied Biosystems,

Foster City, CA) at SB RAS Genomics Core Facility.

### 5BS BAC library screening

To simplify BAC library screening for PCR markers, DNA from 5BS BAC clones were pooled: (i) plate pools containing all 43,776 BACs (384 BACs per pool, 114 pools), and (ii) row and column pools for the constructed physical map. PCR was performed in a reaction mixture (20 μl) containing 2 μl of pooled culture medium, 1 U of DNA *Taq* polymerase, 0.5 μM of each primer, 25 mM of each dNTP, PCR buffer (67 mM Tris–HCl pH 8.8, 18 mM (NH_4_) SO_4_, 1.7 mM MgCl_2_, and 0.01% Tween 20). Amplification was initiated with an initial denaturation of 94 °C for 4 min followed by 35 cycles of 94 °C for 1 min, 50 to 60 °C for 1 min, 72 °C for 1 min, and final stage at 72 °C for 10 min. The resulting fragments were electrophoresed in a 1% agarose gel.

### Construction of genetic linkage maps

The genetic linkage map was constructed with MultiPoint software [34] using 360 genotypes of the F_2_ population from a cross of CS and CS-5Bdic, genotyped with SSR and ISBP markers.

### In silico *fingerprinting of chromosome 5B of T. dicoccoides*

The *.fasta file of the *T. dicoccoides* 5B pseudomolecule was split into 100 kb fragments (analogs to BACs) with a 50 kb overlap. Each such fragment was in silico fingerprinted by LTC software, with parameters used to corresponding in vitro fingerprinting of 5BS (CS). Borders of in silico bands were detected in correspondence to restriction sites of *BamH*I, *EcoR*I, *Xba*I, *Xho*I and *Hae*III enzymes [31]. The lengths of bands (from 50 to 500 bp) corresponding to restriction sites of *BamH*I, *EcoR*I, *Xba*I or *Xho*I, were multiplied by 30. To put all bands on a one-dimensional scale, the resulting numbers were increased 15,000, 30,000 and 45,000 for bands with end(s) corresponding to *EcoR*I, *Xba*I and *Xho*I, respectively. The resulting in silico fingerprints were compared with all in vitro fingerprints from the CS 5BS BAC-library. A significant amount of shared bands (according to Sulston score used for construction of the physical map) enabled the anchoring of ~ 50% of MTP BACs to 100 kb parts of the *T. dicoccoides* 5B pseudomolecule. Although B-genomes of CS and *T. dicoccoides* differ to some extent (about one substitution per 100 bp), most of the substitutions appear in the internal parts of sequences corresponding to the band. Hence, most bands are expected to be shared, enabling the anchoring.

## Results

### Development and anchoring of PCR markers

**Fig. 1:**
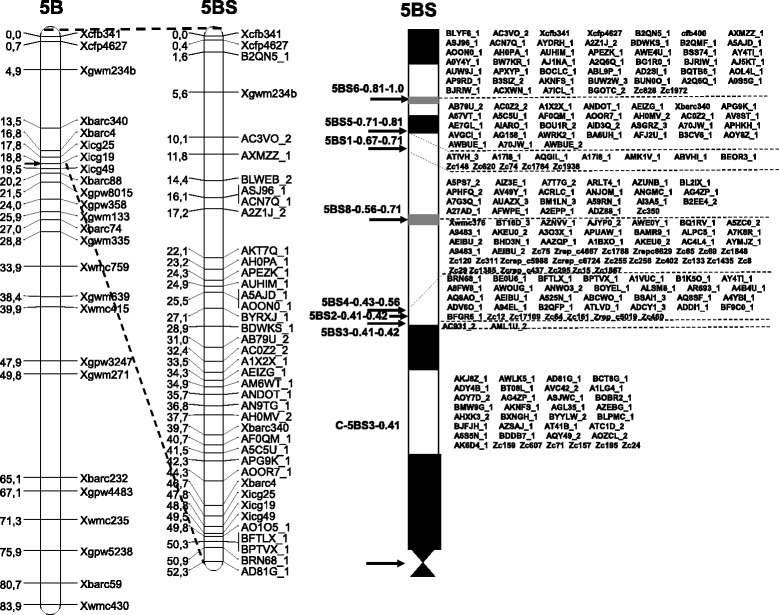
Distribution of SSR, ISBP and Zipper markers along the 5B chromosome**.** Left, genetic map with the distances between markers (cM) and their designations. Right, scheme of the 5BS cytogenetic map; arrows denote boundaries of deletion bins. Bin designations give the information about the arm length they cover; C, centromere. C Banding pattern is given according to http://www.k-state.edu/wgrc/genetic_resources/chinese_spring_deletion_lines.html

To facilitate in vitro anchoring of physical contigs (based on BACs and deletion bins) to genetic maps, we used PCR markers. At the first stage, markers mapped to 5BS (https://wheat.pw.usda.gov/GG3/) were used for this purpose. In total, 123 SSR markers were tested using CS-5Bdic and CS DNA samples in order to detect markers suitable for genotyping 366 individual plants from the F_2_ CS × CS-5Bdic population and deletion lines. This analysis allowed only a limited number of markers (8 for 5BS and 17 for 5BL) to be involved in analysis of the F_2_ population (Fig. 1). Additional markers were generated from 454 GS-Junior sequences that covered ~ 6% of 5BS [21]. We developed 665 ISBP markers (see Materials and Methods). These markers were tested using DNA from CS-5Bdic, CS, N5BT5A, N5BT5D, and Dt5BL; 182 markers were selected and located on the deletion bins (Fig. 1, Additional file 1). Out of them, 32 ISBP markers were found to be polymorphic between CS and CS-5Bdic and were used to construct the 5BS genetic map (Fig. 1). The resulting genetic map was confirmed by the position of markers on deletion bins. Anchored SSR and ISBP markers were used for screening of the chromosome 5BS-specific ВАС library.

### BAC libraries of the 5BS

To construct 5BS-specific BAC libraries, DNA of this chromosome arm was isolated by flow-cytometry sorting. FISH on random samples of sorted chromosome fractions revealed an average purity of 90% for 5BS. A total of 6.8 million 5BS (4.03 μg DNA) were collected. The DNA was used to construct a chromosome arm-specific BAC library TaaCsp5ВShA (43,776 BACs) with average insert sizes of 122 kb. The libraries represent ~ 15.8 equivalents of the estimated sizes of 5BS (290 Mbp [35]). A 5BS-specific BAC library was fingerprinted using SNaPshot technology. Only high quality fingerprints (32,283 5BS-BACs with 18–207 bands) were used to build a physical map.

### Fingerprinting of the 5B chromosome BAC clones and BAC contig assembly

A 5BS-specific BAC library containing 43,776 clones was fingerprinted using SNaPshot technology. A total of 32,283 high quality fingerprints (73.7%) was obtained and used to build a physical map. The first automated assembly was performed with FPC software [29]. This allowed for assemblage of 24,378 fingerprints into 3164 BAC clones, representing 287 Mb (99% of 5BS chromosome arm) with an N50 of 466 kb and an L50 of 159 (Table 1).

**Table 1:** Assembly using FingerPrinted Contig (FPC) and Linear Topological Contig (LTC) software

	FPC	LTC
Useful fingerprints	32,283	32,283
Number of Clones in contigs	24,378	25,857
Singletons	7905	6426
Contig number	763	262
Number of contigs > 5 clones	560	111
^a^Estimated chromosome coverage (%)	99	102
^a^Contig N50 (kb)	466	3078
^a^Contig L50	159	34
Number of clones in MTP	3164	3090

^a^Calculated based on contigs with > 5 clones

The 43,776 clones of the 5BS chromosome-specific BAC library were re-arrayed into two-dimensional BAC pools for library screening. A total of 114 two-dimensional (row and column) pools from the whole 5BS BAC library were produced. Then 20 SSR markers and 45 ISBP markers localized to the 5B chromosome were used to screen the BAC library (Additional file 2). In total, markers were detected in 892 BAC clones (Additional file 3). These data were used for the verification of physical map construction by LTC software.

LTC was specifically designed to construct physical maps for complex genomes, such as that of wheat [30]. To improve the assembly of the 5BS map for future sequencing, we performed an automated LTC assembly using the same 32,283 high quality fingerprints. This resulted in the assembly of 25,857 ВАС clones into 262 contigs, representing 353,931 Mb (122% of the chromosome arm). Contigs with 2–5 clones had much lower coverage (mostly 1 or 2×) relatively to others (having on average coverage 10.4×). We considered these short contigs as questionable (Q). The number of contigs comprising over five clones was 111 (N50 length of 3078 kb and number L50 of 34), and they covered 102% of the 5BS chromosome arm (Table 1, Additional file 2). The number of clones included into MTP (taking into account the contigs comprising over two clones) was 3090. The 2785 BAC clones included into MTP (including the contigs comprising over five clones) were deposited in the URGI site (https://www.wheatgenome.org/content/view/full/574, https://wheat-urgi.versailles.inra.fr/Chr5B).

Using LTC software considerably improved the assembly of BAC clones into contigs as compared with the FPC physical map. The maximum contig size was 13,682 kb in the LTC map, which is 7.7-fold longer than the 1780 kb in the FPC. The L50 value decreased more than 4 times (34 versus 159 for contigs with ≥6 clones). The contig N50 increased more than 6.5 fold for LTC vs. FPC map assembly (Table 1).

**Fig. 2:**
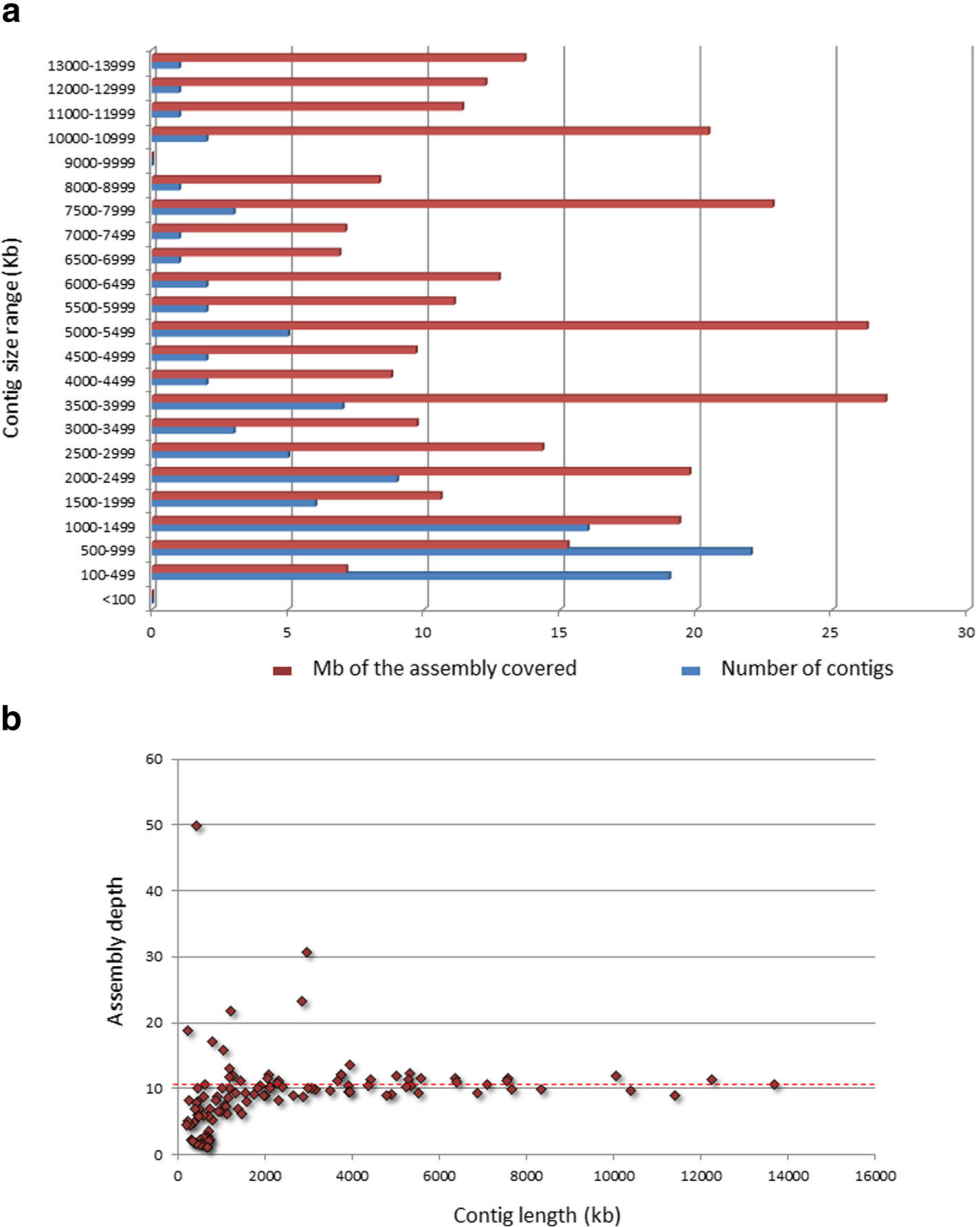
Distribution of assembled contig length. **a** Distribution of assembly coverage across different size ranges. **b** Depth of assembly coverage by contig length

To assess the coverage depending on the contig length, the contig sizes were distributed in the ranges of 500 kb for the contigs smaller than 8000 kb and of 1000 kb for the contigs larger than 8000 kb. The contig lengths were plotted against the number of contigs and the lengths of the assembly covered by the contigs in the corresponding size range (Fig. 2a). The contribution of the ranges to the overall assembly length was not uniform. The maximal length of coverage is also observed for the contigs falling into the length ranges of 3500–3999 kb and 5000–5499 kb and the minimal length, for the contigs shorter than 500 kb.

When assessing the depth of coverage for an assembly, the calculated contig length (the number of clones multiplied by the average insert size ≈ 122 kb) was divided by the actual contig length. As shown in Fig. 2b, contigs with a length of up to 1000 kb clustered around a depth of 1×–10×. The average assembly depth estimated using the calculated assembly length divided by the actual assembly length was 10.4×, around which the large contigs clustered. The following seven contigs exceeding the average assembly depth stay apart: ctg86 (16×), ctg51 (17×), ctg179 (19×), ctg57 (22×), ctg31 (23×), ctg5 (31×), and ctg37 (50×).

The physical map of 5BS is deposited in the IWGSC repository at URGI (http://wheat-urgi.versailles.inra.fr/).

### Construction of the consensus genetic map of chromosome 5B

A consensus genetic map of the 5B chromosome was constructed to localize BAC clones associated with molecular markers. Two populations have been analyzed (see Material and Methods). Construction of the genetic map for F_2_ CS × CS-5Bdic is shown in Fig. 1. Twenty seven SSR markers (17 markers for the short arm, 10 for the long arm) were integrated to the genetic map for the recombinant inbred chromosome lines (RICLs) CS × CS-5Bdic, earlier constructed by genotyping with the help of 15 K Illumina Infinium (Additional file 4).

The consensus map based on the two individual genetic maps (Additional file 4) was constructed using the R package LPmerge [36]. The resulting consensus map contained 421 markers (Additional file 4), including 16 markers common to two maps. The consensus map of the short arm of chromosome 5B was used for anchoring the BAC contigs.

### Anchoring BAC contigs

The contigs were localized on the 5BS chromosome arm via several steps. The first data were obtained after screening of the BAC library using 20 SSR and 45 ISBP markers; of these markers, 12 and 32, respectively, were localized on the 5B consensus genetic map and the remaining markers were on deletion bins (Fig. 1, Additional files 2, 3). Thus, the BAC contigs anchored at the first stage of this work covered 131,447 kb, accounting for 45% of the total length of 5BS.

**Fig. 3:**
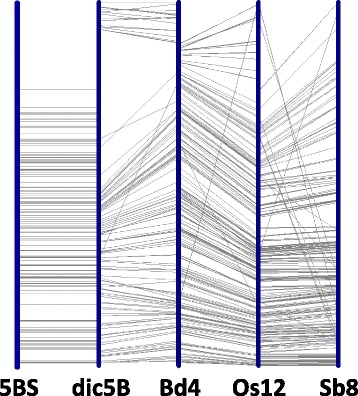
Synteny of wheat (CS) 5BS, wild wheat (*T. dicoccoides*) 5B, Brachypodium Bd4, rice Os12 and sorghum Sb8, based on BLAST. Selected parts are: 0–322 Mb of dic5B, 0–8.1 Mb of Bd4, 8.9–22.9 of Os12, 34.1–55.4 of Sb8. Wheat 5BS is shown schematically (to demonstrate the distribution of markers along dic5B only: no splitting on resulted physical scaffolds/contigs, position of markers does not exactly correspond to physical position on CS 5BS). Markers from CS BLASTed to *T. dicoccoides* are based on BAC-end sequences (e-value cutoff 1e-150). Markers to connect dic5B, Bd4 and Os12 are based on CDS sequences of Brachypodium (e-value cutoff 1e-10). Markers to connect Os12 and Sb8 are based on CDS sequences of rice (e-value cutoff 1e-10)

In order to increase the number of contigs anchored on the chromosome, 134 BAC clones from the middle part of non-anchored contigs were end sequenced and using the Ion Torrent platform. This gave 158 BAC-end sequences and 17,770 Ion Torrent contigs with a total length of 25,879,921 bp [23], which were used further in GenomeZipper analysis. The collinearity regions between the wheat, *Brachypodium,* rice, and sorghum were detected based on local collinearity of their already sequenced genomes related to the wheat genome and the positions of some wheat genes localized earlier on deletion bins [37]. BLASTn (e-value of 1e^− 10^) was used for a comprehensive analysis of the correspondence between wheat, *Brachypodium,* rice, and sorghum. This demonstrated high concordance between the gene order in the corresponding *Brachypodium,* rice, and sorghum regions, collinear to the 5BS chromosome of wheat (Fig. 3). In silico mapping of 17, 770 Ion Torrent contigs of the 5BS arm to the collinear regions of *Brachypodium,* rice, and sorghum made it possible to determine the positions of 50 Ion Torrent contigs on the chromosomes of the studied species. Mapping results of the Ion Torrent contigs on the Bd4:0.9–3.2 Mb region of *Brachypodium* are shown in Additional file 5.

For the Ion Torrent contigs, the markers specific for the studied DNA sequences (referred to as zipper markers; Additional file 6), were designed. These markers, along with SSR markers published previously [23] were used to find correspondence between the Ion Torrent and BAC contigs, as well as to localize the BAC contigs on the genetic and cytogenetic maps (Fig 1). The combination of these approaches made it possible to localize an additional number of BAC contigs and increase the coverage length by 54,527 kb, which together with the first stage covered 185,974 kb. Thus, the coverage of the wheat 5BS arm with anchoring BAC contigs at the second stage of the work attained the level of 64% of the total length of 5BS.

The third approach consisted of comparison of the 5BS BAC fingerprints with in silico fingerprinting of 100-kb fragments of the recently-published *T. dicoccoides* 5B pseudomolecules [14]. Key contigs with positions on the 5B physical map determined by the above described methods were identified. It appeared that the key contigs are located in the same order in the 5BS chromosome arms in *T. dicoccoides* and *T. aestivum* and flank clusters of contigs, while contigs within a cluster can be differently located in *T. dicoccoides* and *T. aestivum* 5BS regions according to the data described above. The high level of similarity in distribution of markers/contigs along 5BS of *T. dicoccoides* and *T. aestivum* was also supported by BAC-end sequences of CS which BLASTed to *T. dicoccoides* (Fig 3). The differences between *T. dicoccoides* and *T. aestivum* observed within some clusters require further study. The use of in silico fingerprinting data for the 5B pseudomolecules of *T. dicoccoides* in combination with the data on anchoring of the BAC contigs obtained by the two approaches described above allowed us to extend the coverage length of the 5B chromosome by 103,922 kb. As a result, the coverage of the total 5BS arm with anchored BAC contigs at the third stage of the work reached 289,896.

**Fig. 4:**
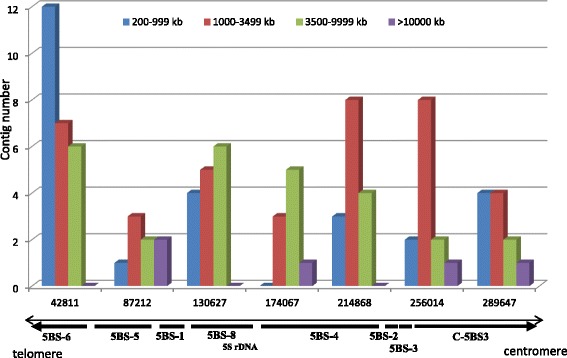
Distributions of contigs of different lengths on the short arm of 5B chromosome. Contigs were divided into 6 approximately equal groups according to the results of the assembly covered by contigs across different size ranges. Anchoring of BAC contigs to deletion bins was carried out by markers mapped in deletion bins or to 5BS genetic maps

The distribution of contigs along the chromosome depending on their length was analyzed (Fig. 4). All contigs formed four groups (200–999, 1000–3499, 3500–9999, and > 10,000 kb) based on the coverage of contig for different size ranges (Fig. 2b). The chromosome was partitioned into six approximately equal intervals (42,000 to 45,000 kb).

Short contigs with a size of 200 to 999 kb were prevalent in the subtelomeric and joined regions (similar to the 1BS chromosome arm, [31]) with a length of up to 42,000 kb. The extended contigs (over 10,000 kb) and the contigs of intermediate length (3500–9999 kb) displayed a uniform distribution in the interstitial region of the 5BS chromosome.

## Discussion

Physical mapping of wheat chromosomes was performed according to the methodology initially designed and later modified by the International Wheat Genomic Sequencing Consortium (IWGSC; http://www.wheatgenome.org), aiming for further use in sequencing and construction of a “gold standard” reference wheat genome, and to provide new recourses for the study of wheat genome organization and evolution, for acceleration of map-based gene cloning and plant breeding.

With the initiation of projects on the generation of high-resolution physical maps in wheat, map-based cloning has become simpler and faster. The number of cloned genes resulting from the use of resources created by physical mapping has grown substantially in recent years [38].

The strategy of high-resolution physical map construction comprises three stages: (1) purification of individual chromosomes or chromosome arms by flow cytometry, followed by construction of chromosome- or arm-specific BAC libraries; (2) high-throughput fingerprinting SNaPshot technology or Whole Genome Profiling [39] and contig assembly; and (3) anchoring of BAC contigs on chromosomes. This strategy was used to construct physical maps for all chromosomes of the wheat genome (http://www.wheatgenome.org) including 5BS, but additional methods and resources were employed at the stage of anchoring of 5BS BAC contigs on the chromosome map.

A comparative analysis of the data on physical mapping versus the year when the corresponding data were published [40–45], including the data described here, is shown in Table 2. It is evident from Table 2 that the use of the LTC program in the assembly of SNaPshot data after the first published physical map for the 3B chromosome considerably improved the quality of BAC clone assembly in contigs. This is emphasized in the published papers for each chromosome, and in particular, is confirmed by the data listed in Table 2. It is also worth mentioning the efficiency using FPC software, which was improved by KeyGene N.V. at processing the WGP data [43]. The contig lengths at N50 varies from 600 to 4000, most likely reflecting the specific features in the organization of individual chromosomes as well as certain variations characteristic to different experimental strategies and protocols; for example, the fingerprinting quality and the number of informative markers used in the screening of BAC libraries (Table 2). In our case, the number of useful fingerprints for the 5BS arm was 32,283 (73.7%) of the 43,776 analyzed BAC clones and the number of the BAC clones selected by the informative markers in screening of the complete library for the short arm was 892 (Additional file 3). As a result, the BAC scaffold length was increased compared to the published physical maps for other wheat chromosomes (Table 2).

**Table 2:** Physical mapping of wheat chromosomes (from published data and the current work)

	3B^a^	1AS^a^	1AL^a^	1BS^a^	1BL^a^	5АS/5AL*^1^	6AS/6AL*^2^	6BS/6BL	3DS	5DS^a^	5BS^a^	5BL^a^
	SNaPshot FTC	SNaPshot LTC	SNaPshot LTC	SNaPshot LTC	SNaPshot LTC	SNaPshot LTC	WPG	WPG FPC	SNaPshot FTC	SNaPshot LTC	SNaPshot LTC	WPG^a^
N50 contig size (kb)	602	798	1166	2430	961	900/601	1090/945	2302/2508	1045	2173	3078	4000
Estimated chromosome coverage^1^%	82%	82%	88%	87%	93%	101%/115%		87%/95%	85.5%	68%	102%	82%
Chromosome size Mbp	960	276	525	314	534	295/532	336/369	415/498	321	258	290	580
References	[8]	[40]	[41]	[31]	[32]	[33]	[42]	[43]	[44]	[45]	This work	URGI site

Size of chromosomes from Safar et al. [35]

^a^Calculated based on an average consensus band length of 1.2 kb (10.1371/journal.pone.0059542.t002) and for contigs > = 6 clones, *^1^ > = 5 clones, *^2^ > = 2 clones

The high level of 5BS BAC contig anchoring (96%) allows us to identify some specific features in the organization of the 5B chromosome when considering the length distribution of contigs along the chromosome. It is noteworthy that the presence of repeated sequences can have a significant effect on the contig assembly length. In particular, gaps between contigs if they are present, tend to overlap with regions of tandem repeats. The analysis of the position of ribosomal RNA genes based on sequencing of the 5B BAC clones showed that the gap between contigs coincides with the region of tandemly repeated 5S RNA genes [46]. Thus, the distribution of short contigs along the chromosome most likely suggests that these regions are enriched for tandem repeats and vice versa, the distribution of longer regions indicates the presence of extended gene-coding regions. A possible exception is the chromosome regions with extended heterochromatin stretches (tandem repeats of different lengths). The tandem repeats residing there escape analysis as early as at the stages preceding the contig assembly for a physical map (BAC clone production and selection of useful fingerprints). The extended heterochromatin regions in the 5B short arm are located closer to the telomere and centromere (Fig. 1). Thus, Fig. 4 shows the distribution of contigs with different lengths localized in between two large heterochromatin blocks at the telomere and centromere. Short contigs (200–999 kb) as markers for the regions interrupted by tandem repeats, are mainly localized to the 5BS6 subtelomeric block, which covers 20% of the short chromosome arm. An analogous pattern, i.e., the localization of short contigs to the subtelomeric chromosome region, was observed in the 1BS chromosome [31]. The distribution of 1000–3500-kb contigs along the chromosome better correlates with the distribution of the regions syntenic to rice, Brachypodium, and sorghum detected by the Zipper approach. The contigs with extended length (more than 10,000 kb) and intermediate length contigs (3500–9999 kb) were uniformly distributed in the interstitial region of the 5BS chromosome.

Interestingly, one contig (ctg37) with a length of 428 kb stands apart when assessing the coverage depth for the 5BS arm in that it exceeds the average assembly coverage more than fivefold (Fig. 2), suggesting that ctg37 belongs to the highly repetitive fraction. It is not improbable that the sequences of ctg37 may be involved in the formation of a heterochromatin block at the boundary of two distal bins (5BS6–5BS5) of the short arm, since this contig resides exactly in this particular region on the physical map.

In general, the construction of the high quality 5BS physical map allowed us to identify some specific features in the organization of the 5B chromosome. Furthermore, the 5BS physical map will support the assembly and annotation of “gold standard” reference sequences for the 5B bread wheat chromosome. The genetic and bioinformatics resources developed in this study provide new possibilities for exploring chromosome organization and for breeding applications.

## Conclusion

The development of physical maps of individual bread wheat chromosomes is an important step in constructing a “gold standard” reference sequence of the wheat genome (the size of individual chromosomes varies from 250 to 960 Mbp).

During physical mapping, different genetic resources were developed which were broadly used in fundamental and applied studies of wheat. This included a large number of markers resulting from BAC-end sequencing and sequencing of BAC clones. The development of BAC libraries of bread wheat as a basis for physical mapping made it possible to get close to the study of the structure and function of a number of genes controlling agricultural traits. This can be clearly seen from the number of genes currently studied for chromosome 3B [38], for which the first stage of physical mapping was completed in 2008. No less important is the development of various deletion and introgression lines and mapping populations, including recombinant inbred chromosome lines (RICL), that allow not only mapping of genes and identifying markers closely segregated with genes, but also direct inclusion in breeding programs.

Construction of the physical map of chromosome 5B was accompanied by the localization of more than 500 markers. BAC clones and markers were involved in the analysis of various regions of chromosome 5BS, including those carrying the genes *SKr, Lr52* and *Yr47* [47, 48]. RICLs created during this work were used for the identification of new heading date determinants in the wheat 5B chromosome [19]. The introgressive bread wheat lines used for cytogenetic mapping of chromosome 5B [20] were then included in breeding programs. Varieties of bread wheat, created with the participation of these lines, are being tested in the field in different regions of Russia.

Thus, despite the laboriousness of the process, the creation of a physical map is important for both fundamental and applied research.

## Additional files

Additional file 1: ISBP and SSR markers based on 454 lower coverage sequencing. (XLSX 21 kb)

Additional file 2: Contig lengths and markers used in BAC library screening. (XLSX 12 kb)

Additional file 3: BAC clones screening. (DOCX 17 kb)

Additional file 4: Consensus 5B map. (X1CSF2 – F2 population from cross Chinese Spring x Chinese Spring-5Bdic; X2CSRICL - RICL from cross Chinese Spring x Chinese Spring-5Bdic). (XLSX 21 kb)

Additional file 5: Mapping of IonTorrent contigs on Brachypodium region (Bd4: 0.9–3.2 Mb). (XLSX 16 kb)

Additional file 6: Design of primers for zipper (zc) markers. (XLS 40 kb)

## Abbreviations

5BS: Short arm of wheat chromosome 5B
BAC: Bacterial artificial chromosome
CS: Chinese Spring
CS-5Bdic: Disomic substitution line, where the CS 5B chromosome is replaced by the *T. dicoccoides* 5B chromosome (5Bdic)
FPC: FingerPrinted Contigs
ISBP: Insertion site–based polymorphism
IWGSC: International wheat genome sequencing consortium
LTC: Linear TOPOLOGICAL Contig
RICLs: Recombinant inbred chromosomal lines
SSR: Simple sequences repeats
