# Supplementary material for: Features of the organization of bread wheat chromosome 5BS based on physical mapping
Source: BMC Genomics. 2018 Feb 9;19(Suppl 3):80. doi: 10.1186/s12864-018-4470-y (PMC5836826; doi:10.1186/s12864-018-4470-y)
Supplement: Supplementary file 3 — BAC clones screening. (DOCX 17 kb) [file 12864_2018_4470_MOESM3_ESM.docx]

Table 2S. BAC clones screening

| Type of marker | Number of markers | Number of clones |
| --- | --- | --- |
| **SSR** located in bins, | **20** | **120** |
| from them - located in genetic map | 12 | 97 |
| **ISBP** located in bins | **45** | **772** |
| from them located in genetic map | 32 | 575 |
| **Total** | **65** | **892** |
